# Disaster mycology

**DOI:** 10.7705/biomedica.6943

**Published:** 2023-08-31

**Authors:** Daniel F. Q. Smith, Arturo Casadevall

**Affiliations:** 1 W. Harry Feinstone Department of Molecular Microbiology and Immunology, Johns Hopkins Bloomberg School of Public Health, Baltimore MD, USA School of Public Health Baltimore MD USA

**Keywords:** Mycology, fungi, climate change, Candida auris, natural disasters., micología, hongos, cambio climático, Candida auris, desastres naturales.

## Abstract

Natural and human-made disasters have long played a role in shaping the environment and microbial communities, also affecting non-microbial life on Earth. Disaster microbiology is a new concept based on the notion that a disaster changes the environment causing adaptation or alteration of microbial populations-growth, death, transportation to a new area, development traits, or resistance-that can have downstream effects on the affected ecosystem. Such downstream effects include blooms of microbial populations and the ability to colonize a new niche or host, cause disease, or survive in former extreme conditions. Throughout history, fungal populations have been affected by disasters. There are prehistoric archeological records of fungal blooms after asteroid impacts and fungi implicated in the fall of the dinosaurs. In recent times, drought and dust storms have caused disturbance of soil fungi, and hurricanes have induced the growth of molds on wet surfaces, resulting in an increased incidence of fungal disease. Probably, the anticipated increase in extreme heat would force fungi adaptation to survive at high temperatures, like those in the human body, and thus be able to infect mammals. This may lead to a drastic rise of new fungal diseases in humans.

## Resumen

Los desastres naturales o los causados por el hombre impactan la formación de ecosistemas y comunidades microbianas, y también afectan las formas de vida no microbianas. Este concepto es conocido como “microbiología de desastres”, una subespecialización de la microbiología, basada en los cambios ambientales generados por un desastre y las posibles adaptaciones o alteraciones de las poblaciones microbianas -crecimiento, muerte, trasporte a una nueva región, o adquisición de resistencia o de nuevas características- que influirán en el moldeamiento del ecosistema transformado. Algunos de los efectos de estas adaptaciones pueden ser: el surgimiento de poblaciones microbianas, la habilidad de colonizar nuevos nichos u huéspedes, la generación de nuevas enfermedades, o el crecimiento de microorganismos en condiciones que antes eran “extremas” para ellos.

A lo largo de la historia, varias poblaciones de hongos han sido afectadas por desastres. Existen registros arqueológicos prehistóricos que evidencian la presencia y el crecimiento de hongos luego del impacto de asteroides, y otros de hongos relacionados con la extinción de los dinosaurios. Actualmente, las sequías y las tormentas de polvo causan perturbaciones en las comunidades de hongos del suelo, y los huracanes inducen el crecimiento de hongos filamentosos en superficies húmedas, lo que aumenta la cantidad de enfermedades por hongos. Además, con el aumento de las temperaturas extremas es posible que los hongos puedan adaptarse para sobrevivir a temperaturas más altas, equivalentes a las temperaturas corporales, y nuevas especies puedan infectar mamíferos. Esto puede llevar a un aumento drástico de las infecciones fúngicas en humanos.

Disaster microbiology is a newly described field that seeks to understand how natural disasters (such as hurricanes, tornadoes, tsunamis, droughts, and extreme heat) and human-made disasters (such as pollution, war, mechanical failures, and nuclear reactor meltdowns) affect microorganisms, and how these microbial changes affect ecosystems and human health ^1^^,^^2^. We could define disaster mycology as the study of how natural and human- made disasters impact fungal populations, for example, by affecting fungal properties such as stress adaptations, virulence, and their growth in new niches and locations that could impact human, animal, and plant health.

## Prehistory

Fungal communities have been directly impacted by natural disasters for hundreds of millions of years. The first fossil records of fungi date from at least 800 million years ago to potentially as far back as 2.4 billion years ago ^3^. Fossils of filamentous microbes, consistent with fungi, are growing on an impact crater dating to 458 million years ago during the Ordovician period ^4^. It is possible that this fungal colonization, following the meteor impact, occurred due to the heat and hydrocarbons provided by the impact and the resulting hydrothermal system. This is an early example in which a natural disaster permitted the growth and colonization of a fungal species in a new environmental niche.

Following the largest extinction event in global history, the Permian- Triassic extinction, or the “Great Dying,” approximately 252 million years ago, there was an apparent surge of fungal growth. Evidence for this reported fungal growth comes from a fossil record of *Reduviasporonites* spores, a layer with a thickness ranging from centimeters to almost a meter, containing scarce other organic matter or fossils ^5^^,^^6^. However, this interpretation of the fossil record remains controversial due to physical and biochemical similarities between algal and fungal spores ^7^. Other hypotheses propose that these fungal species increased in prevalence due to the massive amount of decaying biomass following the extinction event ^8^; or that the ecological shifts following volcanic eruptions and drought during the Permian extinction event weakened the immune systems of plants and made them more susceptible to infection with *Reduviasporonites*, as occurs with modern plants following natural disasters, resulting in the loss of forests due to fungal disease ^8^. Gueidan *et al*. molecularly and paleontologically dated the origin of rock-inhabiting fungi to the Triassic period during and after the Permian extinction ^9^. This mass extinction event may have reduced competition for slow-growing rock-inhabiting fungi ^9^, the formation of the landmass Pangea caused widespread and prolonged droughts, spurring the evolution of relatively stress-resistant fungi that inhabited rocks and were capable of growing in nutrient-poor conditions ^9^. The ancestors of these rock-inhabiting fungi are still found in nutrient-poor ecosystems today.

Similarly, the geological boundary that corresponds to the time between the Cretaceous and Tertiary periods, 65 million years ago, known as the K-T boundary, contains a layer of soot and fungal spores ^10^. The spike of fungal spores coincides with the extinction of dinosaurs, and an iridium spike suggests a meteor collision, likely at the Chicxulub impact, off the coast of what is now the Yucatan peninsula. Together, these features paint a picture of a meteor impact causing a massive loss of plants and animals, which resulted in a proliferation of fungi as decomposers. Such findings have also led to a hypothesis that the fungal bloom after the massive ecosystem disruption, and the death at the end of the Cretaceous, conduced to the death of surviving dinosaurs on Earth and the survival and proliferation of mammals due to its resistance to fungi ^11^^-^^13^.

This hypothesis, named Fungal-Infection Mammalian-Selection posits that the global cooling following the K-T cataclysm affected dinosaurs’ thermoregulation, causing them to suffer cooler body temperatures than expected. Dinosaurs were supposed to be mesotherms, meaning their body temperatures varied according to body size, baseline metabolic rate, and environmental conditions. These conditions altered the dinosaurs’ control over their body temperature, unlike mammals who are endothermic ^14^^,^^15^. As a result, smaller dinosaurs and dinosaurs in colder climates may have had lower baseline body temperatures ^14^^,^^15^.

These body temperatures, influenced by post-disaster famine and cooling conditions, could have led to fungal proliferation. Most known modern-day fungi do not survive at higher body temperatures (greater than 37 °C), which provide a natural resistance to fungal infection ^16^. That temperature barrier blurring could have predisposed dinosaurs to fungal disease ^11^. Bony lesions consistent with chronic fungal disease have recently been described in dinosaurs’ bone fossils ^17^. However, whether fungal diseases contributed to the demise of the dinosaurs is unknown. The ecological catastrophe associated with the meteorite impact may have been sufficient to eventually cause dinosaurs to go extinct. The findings of a fungal bloom following the calamity would have exposed any survivors to large *inocula* of fungal spores, placing an additional stress on those remaining alive. Conversely, the Fungal- Infection Mammalian-Selection hypothesis proposes that mammals, which can retain their body temperature homeostasis better than large reptiles, survived and became a dominant class of life on Earth because their temperatures protected them against fungal diseases after the K-T boundary calamity.

The complete effects of such prehistoric disasters on fungal populations, and thus on global ecosystems, on plant or animal health, or on the course of fungal evolution, are hard to estimate. Theories can be suggested from fossil records, isotropic analysis, and genetic histories, but is unknown the extent of the catastrophe’s consequences for mycology.

## Recent history

Natural disasters have affected fungal populations since the end of the age of dinosaurs, through the age of mammals, and throughout human civilization. However, detailed mycological effects of disasters have rarely been described. Some epidemiologists suggested that the tenth plague described in the Book of Exodus, death of the first-born son, is actually related to disaster mycology ^18^^,^^19^. They hypothesized that the hail from the seventh plague would have destroyed crops, causing moldy wheat and mycotoxin production. The ingestion of toxins induced illness and death. Beyond using fungal agents to interpret historical or religious events, there are scarce examples tracing the fungal adaptations or alterations as consequence of disasters. Until recent centuries, fungal diseases began to be recognized, diagnosed, and named.

## Disasters, changes in host susceptibility, and the rise of fungal diseases

The second half of the 20^th^ century ushered in an age of fungal diseases unprecedented in human history, partly due to the erosion of the immune system in some populations ^20^. Specifically, those decades saw the rise of the HIV/AIDS epidemic, proliferation of immunosuppressive anti-cancer chemotherapies, and immunosuppressive steroids used to treat transplant recipients and patients with other chronic and inflammatory diseases. Some of these conditions may arise in part from disasters. For instance, the human- made disaster of air pollution, with petrochemicals and particulate matter, resulted in elevated rates of lung cancer and acute myeloid leukemia ^21^^-^^23^. Exposure to a human-made radiation disaster, such as the fallout following the atomic bombings in Hiroshima and Nagasaki, increased the incidence of acute myeloid leukemia in the population ^24^. Rates of acute myeloid leukemia and other hematological malignancies intensified for people who lived near the site of the Chernobyl nuclear power plant disaster in Ukraine or helped in decontamination efforts ^25^^,^^26^.

Treatment for those cancers involves immunosuppressive immunotherapies, increasing the risk of fungal disease development, while acute myeloid leukemia results in immunosuppression because it affects all the myeloid-derived blood cells, including important immune cells during fungal infections such as macrophages, monocytes, and neutrophils. Additionally, acute myeloid leukemia can be treated with bone-marrow transplantation accompanied by a previous corticosteroid treatment to prevent transplant rejection, but this can cause additional immunosuppression. Among patients with hematological malignancies, up to 4% develop invasive fungal infections ^27^. A study about the prevalence of fungal infections in hospitalized children with malignancies found that up to 7% developed fungal infections, predominately associated with acute myeloid leukemia and neutropenia ^28^. Natural disasters are defined by the United States Federal Emergency Management Agency (FEMA) as, “the negative impact(s) following an actual occurrence of natural hazard(s) in the event that it significantly harms a community ^29^.” While FEMA does not list pandemics, epidemics, or plagues as a natural hazard or natural disaster, De Rubeis *et al*. have recently posited that pandemics and epidemics fall under the category of natural disaster as they arise from a natural origin and cause sudden, significant, and multifaceted harm to communities ^30^.

With this in mind, we would consider the HIV/AIDS epidemic and the continued spread of HIV (Human Immunodeficiency Virus) as a natural disaster. An HIV-untreated infection could result in the loss of CD4+ T-cells, and ultimately, in a severely immunocompromised status. In this state, individuals can develop opportunistic infections and disease manifestations linked to the acquired immunodeficiency syndrome (AIDS). Many common opportunistic infections are invasive fungal infections caused by *Pneumocystis jirovecii*, *Cryptococcus neoformans, Candida albicans*, or Histoplasma spp. Out of early 398 AIDS-related deaths in the United States, 240 occurred in patients infected exclusively with *P. jirovecii*, before the introduction of effective antiretroviral therapies ^31^. In recent estimates, approximately 50% of AIDS-related deaths were due to invasive fungal infections, mainly in patients not undergoing highly active retroviral therapy, with nearly one million deaths occurring annually ^32^.

Lately, with the emergence of SARS-CoV-2 in late 2019 and the subsequent COVID-19 pandemic, we saw an additional instance where a pandemic disaster resulted in an increase of fungal infections. Fungal superinfections have been noted in patients following severe COVID19 cases, particularly those caused by *Aspergillus, Rhizopus, Cryptococcus*, and *Candida species*^33^^,^^34^. Cases of invasive fungal infection after severe COVID-19 are associated with certain risk factors, including immunosuppressive corticosteroid treatments to treat severe symptoms and inflammatory immune responses, prolonged ventilation and intubation, and underlying conditions such as cancer ^34^. In intubated COVID-19 patients, the frequency of COVID-associated pulmonary aspergillosis was 2-15% ^35^. Since the beginning of the COVID19 pandemic in the United States, fungal infections death rate was from 1.2 per 100,000 people per year to 1.8 per 100,000 per year ^36^. Additionally, 21.9% of deaths caused by fungal infections in 2020-2021 in the United States were associated with COVID-19 infections ^36^.

### *Disasters and alterations to fungal communities in the 20* ^*th*^ *and 21* ^*st*^ *centuries*

While some examples of pollution, radiation, war, and pandemic disasters have eroded the host immune response and made people more susceptible to fungi, other disasters have altered fungi in a way that puts them in direct contact with people, thus causing disease. For example, during earthquakes, dust storms, and droughts in the American Southwest, *Coccidioides* spp. can become aerosolized from displaced soil and inhaled by people in the affected areas. These resulted in localized outbreaks of *coccidioidomycoses*, in the cases of a windstorm in California in 1977, the Northridge Earthquake in California in 1994, and repeated droughts in Arizona throughout the late 1990s ^37^^-^^39^. Similarly, natural disasters such as tornadoes, like the ones occurring in Joplin, Missouri, in 2011, can cause severe wounds that become infected when exposed to environmental fungi in debris and the air, leading to serious fungal infections such as mucormycosis ^40^. In other instances, floodings, such as occurred during the East Japanese tsunami of 2011 and the Indian Ocean tsunami of 2004, also produce the aerosolization of soil and ground fungi, provoking “tsunami lung”, which consists of severe fungal infections like aspergillosis, scedosporiosis, and mucormycosis ^41^^,^^42^.

In other instances, natural disasters can shift the environmental niches of fungi and lead to their growth in new areas, causing new exposure and subsequent disease. For example, a hypothesis stated that the Great Alaskan Earthquake of 1964 followed by a tsunami in the Pacific Ocean caused fungi, such as *Cryptococcus gattii*, to wash ashore and colonize the land of the Pacific Northwest of the United States and Canada, including Vancouver Island ^43^. The phylogenetic analysis shows a divergence of the Pacific Northwest environmental samples, approximately between 1930 and 1950 and an association between the tsunami-struck location and the incidence of *C. gattii* infections and its environmental isolations. The *C. gattii* that may have colonized the Pacific Northwest coast has caused periodic *C. gattii* outbreaks since 1999 in seemingly immunocompetent individuals ^43^^,^^44^. Other flooding events, including hurricane Katrina in 2005 and hurricane Maria in 2017, provoked water damage to structures, which induced new fungal growth, usually in the form of mold ^45^^-^^47^. This new mold growth can generate serious health issues for residents, including asthma, sick building syndrome, or other allergic reactions. Following hurricane Harvey in 2017, invasive mold infections in Houston, Texas, increased even in persons who would not typically be considered at risk for invasive fungal diseases ^48^.

In the 20^th^ and 21^st^ centuries, we have seen instances of fungal infections following human-made or hybrid disasters. In 1997, during the Maccabiah Games in Israel, the Maccabiah Bridge spanning the Yarkon River collapsed while the athletes of the Australian delegation were crossing. This river was heavily contaminated with waste and pollution ^49^. Following the collapse, several members of the delegation inhaled aerosolized *Pseudallescheria* boydii (*Scedosporium apiospermum*) fungus, resulting in systemic infections and death of four individuals ^49^^,^^50^. Other instances of fungal infections have arisen from human-caused disasters in the form of contaminated medical supplies. Following the 2004 Indian Ocean tsunami, some medical supplies, including spinal anesthesia, were kept in sub-optimal conditions. Anesthesia supplies became contaminated with Aspergillus fumigatus, which resulted in an outbreak of fungal meningitis in women who underwent caesarean section surgery ^51^. In 2012-2013, several batches of preservative-free methylprednisolone, typically injected epidurally, were contaminated by the fungus *Exserohilum rostratum* due to improper sterilization and unsafety protocols by the manufacturing company. This contamination led to an outbreak of 749 cases of *E. rostratum* infection, including 391 with fungal meningitis, resulting in approximately 40 deaths ^52^^,^^53^. In Summer and Fall 2022, there were similar cases of fungal meningitis in Durango, Mexico: the use of epidermal anesthesia contaminated with the fungus *Fusarium solani* caused 77 cases and 29 deaths ^54^.

## The future

The past century saw the rise of fungal diseases in humans, primarily due to alterations in the fungal environment and their hosts, including the weakening of our immune systems through immunosuppressive medical treatments, the HIV/AIDS epidemic, or fungal exposure following disasters. As climate change accelerates, in the next century and beyond, without mitigation efforts, we can expect increasing flooding disasters, droughts, extreme heat and heatwaves, and increased emergence and incidence of pandemic or epidemic-capable pathogens ^20^^,^^55^^,^^56^. These disasters, caused by anthropogenic climate change, are predicted to cause shifts in the environmental niches of some fungi, via drought and flooding ^57^, while other disasters, including extreme heat, can drive the adaptation of fungi to survive at human body temperature and thus become potential pathogens ^58^^,^^59^.

### Fungal adaptations to a changing climate

One feature of mammals, including humans, is endothermy. It allows us to maintain a high body temperature, which has protected us from being infected by many fungal species, even during immune deficiencies ^16^. However, with increasing global temperatures and more frequent and longer duration of extreme heat events, it is hypothesized that fungi will gain thermotolerance and be able to persist at human physiological body temperature ^59^^,^^60^. The adaptation to extreme heat in the coming decades may result in the emergence of more fungal species capable of causing disease in humans. Analysis of the thermotolerance of fungal species deposited in the Westerdijk Fungal Biodiversity Institute collection showed an increase in the maximal growth temperature for basidiomycetous yeasts over recent decades, consistent with adaptations to a warming climate ^61^. It may have already occurred with *Candida auris*, first recognized clinically in 2009, and genetically distinct clades described almost simultaneously across the globe ^58^. The isolates of C. auris recovered during recent outbreaks of the fungus appear to have diverged within the past 40 years, in line with the rising global temperatures ^62^, while closely related fungal species do not share the same degree of thermotolerance as *C. auris*^58^. *C. auris* poses an ominous threat, not only due to its durability and high resistance to drugs and environmental stress, but also because it may foretell what the 21^st^ and 22^nd^ centuries’ infectious disease landscape has in store.

## Alterations to fungal ecosystems

Heat and climate change also can result in droughts and changing of ecosystem boundaries. Some prediction affirms that the increased frequency of drought and dust-storm conditions in the American Southwest could dramatically extend the geographical distribution of Coccidioides, increasing the risk of millions of people suffering coccidioidomycosis in the coming decades ^57^^,^^63^. Similarly, changing weather patterns can modify the geographical ranges of animal reservoirs and vectors carrying microorganisms and viruses that can cause diseases ^55^^,^^56^, potentially leading to new epidemics or pandemics. As we have seen with the HIV/AIDS, influenza ^64^, and COVID-19 public health disasters, even the spread of non-fungal pathogens can lead to fungal infections burgeoning and disease. Lastly, in the 21^st^ century, tropical cyclones are expected to increase in number and intensity as ocean temperatures rise ^65^^,^^66^. These flooding disasters increase the risk of mold damage, resulting in allergic diseases like asthma and overloading the healthcare system with infectious diseases sick people. Additionally, as in the case of C. colonization of the Pacific Northwest, described above ^43^, as the frequency and intensity of floodings continue, it could result in the colonization of land with marine-dwelling fungi. These aquatic fungi may have the ability to cause disease in humans or develop adaptations over time that enable virulence.

## Concluding remarks

Throughout time, disasters have impacted fungi and have caused them to adapt to new environments and inhabit new niches. Following global cataclysms and mass extinctions, fungi flourished ^6^^,^^10^, which could have led to the extinction of the Age of Reptiles ^11^, but also helped recycle nutrients in the environment and re-established life itself. In the past century, natural and human-made disasters have triggered isolated and worldwide outbreaks of fungal infections. In the coming century, as disasters continue to intensify and ecosystems shift due to climate change, we can expect fungal outbreaks, and emerging fungal diseases to likewise intensify and shift. As disasters continue to destroy, fungi will continue to adapt, helping break down ecosystems and rebuilding them. The stories of fungi include disasters, but also stories of recovery.
